# Modulation of gut microbiome in response to the combination of *Escherichia coli* Nissle 1917 and sugars: a pilot study using host-free system reflecting impact on interpersonal microbiome

**DOI:** 10.3389/fnut.2024.1452784

**Published:** 2024-10-22

**Authors:** Kiran Heer, Manpreet Kaur, Dwinder Sidhu, Priyankar Dey, Saumya Raychaudhuri

**Affiliations:** ^1^Molecular Biology and Microbial Physiology Division, CSIR-Institute of Microbial Technology, Chandigarh, India; ^2^Academy of Scientific and Innovative Research (AcSIR), Ghaziabad, India; ^3^Department of Biotechnology, Thapar Institute of Engineering & Technology, Patiala, Punjab, India

**Keywords:** *Escherichia coli* Nissle 1917, probiotic, prebiotic, synbiotic, microbiome, interindividual variation

## Abstract

**Introduction:**

The differential effects of probiotic, prebiotic, and synbiotic formulations on human health are dictated by the inter-individual gut microbial profile. The effects of probiotics such as *Escherichia coli* Nissle 1917 (ECN) on gut microbiota may vary according to the microbiome profiles of individuals and may be influenced by the presence of certain carbohydrates, which can impact microbial community structure and treatment results.

**Method:**

Processed fecal samples from donors having contrasting lifestyles, dietary patterns, and disease histories were mixed with 5 × 10^6^ CFU/mL ECN with or without 1% (w/v) sugars (glucose, galactose, or rice starch) in a host-free system. Post-incubation, 16 s rRNA sequencing was performed. Microbial diversity and taxonomic abundance were computed in relation to the probiotic, prebiotic, and synbiotic treatment effects and interpersonal microbiome variance.

**Result:**

Baseline gut microbial profiles showed significant inter-individual variations. ECN treatment alone had a limited impact on the inter-personal gut microbial diversity and abundance. Prebiotics caused a substantial enrichment in Actinobacteria, but there were differences in the responses at the order and genus levels, with enrichment shown in *Bifidobacterium*, *Collinsella*, and *Megasphaera*. Subject B exhibited enrichment in Proteobacteria and Cyanobacteria, but subject A showed more diversified taxonomic alterations as a consequence of the synbiotic treatments. Despite negligible difference in the *α*-diversity, probiotic, prebiotic, and synbiotic treatments independently resulted in distinct segregation in microbial communities at the *β*-diversity level. The core microbiota was altered only under prebiotic and synbiotic treatment. Significant correlations primarily for minor phyla were identified under prebiotic and synbiotic treatment.

**Conclusion:**

The interindividual microbiome composition strongly influences the effectiveness of personalized diet and treatment plans. The responsiveness to dietary strategies varies according to individual microbiome profiles influenced by health, diet, and lifestyle. Therefore, tailored approaches that consider individual microbiome compositions are crucial for maximizing gut health and treatment results.

## 1 Introduction

Probiotics are living bacteria that, when given in sufficient amounts, provide health advantages to the host, including prophylaxis against chronic diseases (1), through mechanisms involving maintaining of gut microbial community homeostasis and preventing overgrowth of pathobionts (2), enhance the integrity of the intestinal barrier (3), boosting immunological functions (3), and production of beneficial metabolites (4). Nevertheless, reports of conventional probiotic mediated causation of opportunistic infection are widespread (5, 6). Thus, novel probiotic microorganisms are required to address limitations such as low viability during gastrointestinal (GI) passage, strain-specific impacts, and the risk of developing antibiotic resistance (7, 8). Collectively, the objective of the next-generation probiotic science is to identify resilient strains that provide wider and more reliable health advantages, improved durability, and safety, therefore guaranteeing efficient and dependable probiotic treatments for various populations.

*Escherichia coli* Nissle 1917 (ECN), a non-pathogenic strain belonging to the class *γ*-proteobacteria, is a probiotic that is particularly notable for improving gut health and addressing GI diseases. ECN was first isolated by German bacteriologist Alfred Nissle in 1917 (9). ECN has been intensively studied for preserving intestinal barrier integrity (10), regulating the immune system (11), and fostering a beneficial equilibrium of gut microbiota (12). Its GI efficacy has been reported against ulcerative colitis (13), acute diarrhea (14), diabetes (15) and fatty-liver disease (16), and other medical conditions. In line, genetically engineered ECN, capable of producing cholera autoinducer-1 protein, was able to restrict GI colonization of *Vibrio cholerae* and limit its virulence (17). Similar effects were reported against the colonization of *Salmonella typhimurium* (18) and *Pseudomonas aeruginosa* (19) which were likely mediated by the potent iron chelation properties of ECN (20).

It has been established that the beneficial health effects of drugs or diet are strongly influenced by the interindividual variations of the gut microbiome. Indeed, inter-personal gut microbial profile has been demonstrated to influence the bioactivities and bioavailability of drugs and therapeutics (21–24). Moreover, these variations could also be associated with disease susceptibility (25) and therapeutic efficacies of treatment (26). The gut microbial profile has been associated with the health outcomes of nutrients, an essential mediator in the development of precision nutritional strategies (27). Indeed, using *Lactobacillus helveticus* and *Streptococcus thermophilus*, it was demonstrated that the efficacy of probiotics is dependent on the gut microbial profile of individuals (28). Others have shown that the inter-individual gut microbial makeup influence the colonization and efficacy of probiotics (29), influence metabolism and bioavailability of micronutrients (30, 31) and susceptibility to chronic disease (32). Therefore, utilizing the gut microbiota to forecast personalized health impacts to nutritional, prebiotic, and probiotic therapies is essential as it allows for customizing interventions based on an individual’s own microbiome profile, resulting in improved and optimal health outcomes. Although ECN has shown encouraging therapeutic effects, the effectiveness of probiotic therapy might vary significantly across individuals. Interpersonal variations in gut microbiota may impact the colonization and interaction of probiotics with natural microorganisms and their ability to produce positive effects. Hence, it is essential to comprehend the significance of the initial gut microbiota configuration in influencing the response to ECN to enhance probiotic therapies.

In this work, we investigated the notion that the manipulation of the gut microbiota by ECN relies on the individual’s microbiome profile and may be further affected by the introduction of certain prebiotics and associated synbiotics. This was based on the hypothesis that pre-existing inter-individual variations due to lifestyle factors likely influence the impact of the prebiotic and probiotic treatments on the overall gut microbiome shift of an individual. For this purpose, we utilized a simplified approach of a host-free anaerobic culturing system, where fecal slurries from two distinct volunteers were subjected to treatment with ECN alone (i.e., probiotic), individual sugars (i.e., glucose, galactose, and starch) alone (i.e., prebiotic), or combinations of these sugars with ECN (i.e., synbiotic formulation). Similar *in vitro* models of microbiome research have been previously utilized in food, nutrition, and pharmacological research to study the host-independent interaction with the gut microbiota (33–36). Indeed, this is important since the extent of the dynamic nature of the human gut microbiota is strongly dictated by host-specific factors (e.g., diet, age, disease). Thus, removal of the host factor is expected to eliminate the influence from confounding factors. The selection of ECN was in line with our prior *in vitro* study demonstrating that in the presence of sugars, especially glucose, ECN can restrict the growth of *Vibrio cholerae* by producing acidic metabolites (37). Similar results were obtained in the zebrafish model, demonstrating that ECN can reduce inflammation and GI tissue injury that was otherwise induced by the GI colonization of adherent-invasive *Escherichia coli* (AIEC) (38, 39). Although small sample size likely impacted the outcomes in terms of microbial diversity and the identification of core microbiome, the data reflected how inter-individual microbiome profile is important to achieve desired benefits under specific treatments. Collectively, our present study highlights the changes brought about by the exogenous administration of carbohydrates and probiotics on the structure of anaerobic microbial communities in fecal slurries obtained from two donors, emphasizing the fact that the changes on the community structure are also dependent on the existing diversity of the host gut microbiome.

## 2 Materials and methods

### 2.1 Ethical clearance and collection of metadata

Study approval was obtained from the Institutional Ethical Review Committee (September 2018;#2), and experiments were performed following established guidelines and regulations. Written consent was obtained from volunteers prior to collection of fecal matter. Metadata was obtained from each volunteer in the form of questionnaire recording their age, gender, food habit, medication history and health status at the time of sample collection.

### 2.2 Construction of *Escherichia coli* Nissle 1917 rifampicin variant

ECN was sub-cultured with increasing concentrations of rifampicin to generate a rifampicin-resistant mutant. Passaging of bacterial culture was done in 5–75 μg/mL of rifampicin (40). ECN/Rif mutant was then maintained on LB/Rif (75 μg/mL) plates and further confirmed by sequencing. In order to check the survival of ECN/Rif in fecal slurry, after 12 h of incubation, 1 mL of fecal slurry was collected from serum bottle and diluted up to 10^−6^ dilution and then spotting test was performed on LB/Rif (75 μg/mL) plates.

### 2.3 Experimental setup

**Figure 1:**
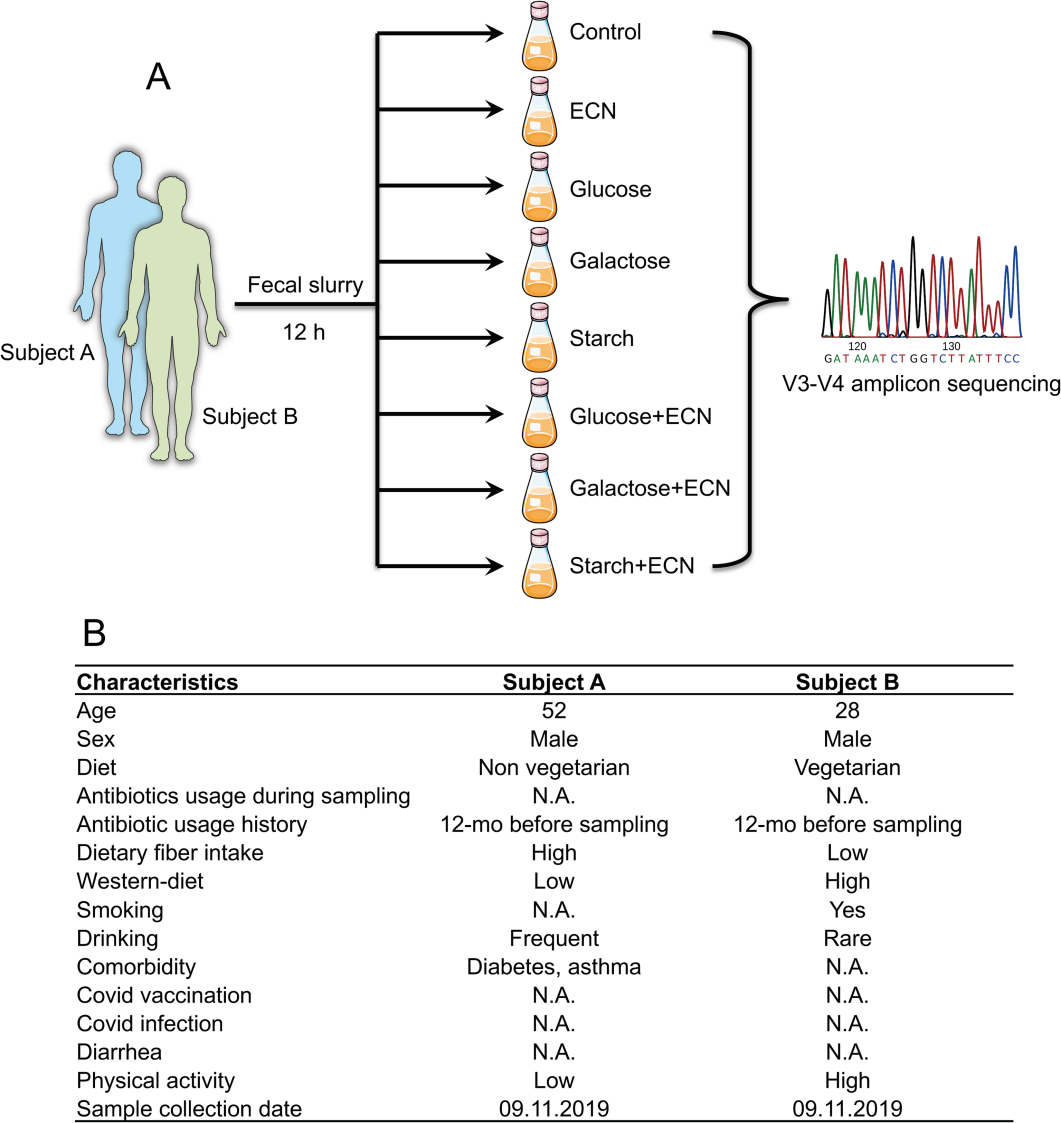
**(A)** Experimental design of the study. Fresh fecal matter was collected from two individuals and prepared fecal slurry was treated with either ECN (probiotic); glucose, galactose or starch (prebiotic); or ECN in combination with glucose, galactose or starch (synbiotic). After 12 h incubation under anaerobic conditions, samples underwent V3-V4 amplicon sequencing of 16S rRNA. Fecal slurries from both individuals were not pooled and underwent separate treatments. **(B)** Characteristics of subjects from whom fecal matter was collected. Abbreviation: ECN, *Escherichia coli* Nissle 1917.

Morning fresh fecal samples were collected from consent donors (self-collection) in sterile containers. Samples were processed immediately to prevent unwanted loss of viability of obligate anaerobes, by preparing a stock of 20% fecal slurry (w/v) in 50 mM PBS buffer following a published protocol (40, 41). The fecal slurries were diluted to 10% in 50 mM PBS. 50 mL of 10% slurries were distributed in a serum vial and mixed with exponentially grown 5 × 10^6^ ECN and 1% (w/v) sugars [D-(+)-glucose (Sigma, G7021), D-(+)-galactose (Sigma, G5388), rice starch (Sigma, S7260)], both alone and in combination (Figure 1A). Dose selection was based on our prior studies demonstrating that a synbiotic formulation comprising 5 million ECN and 1% glucose was effective in controlling *V. cholerae* infection and GI overgrowth of adherent-invasive pathogenic *E. coli* (37–39). Serum vials were purged with an anaerobic gas mixture, sealed, and incubated for 12 h at 37°C at 150 rpm. All procedures were performed inside anaerobic chamber. After incubation, vials were stored for 12 h in −80°C before processing for DNA extraction.

### 2.4 Metagenomic sequencing

After incubation, samples were shipped to Medgenome Labs Ltd. under cold conditions for further processing. In brief, total DNA was isolated using QIAamp Fast DNA Stool Mini Kit (Qiagen; Hilden, Germany). DNA quality was checked using gel electrophoresis and concentration was measured spectrophotometrically using nanodrop (Thermo). DNA samples (5 ng/μL) were subjected to PCR amplification using 515F (5’-GAGTGCCAGCMGCCGCGGTAA-3′) and 806R (5’-ACGGACTACHVGGGTWTCTAAT-3′) primers specific for V3-V4 hypervariable region of the bacterial 16S rRNA to prepare amplicon libraries. NEBNext® Multiplex Oligos for Illumina® (96 Index Primers)—NEB #E6609L was used for library preparation. Individual libraries were pooled at equimolar proportions and subjected to sequencing using Illumina Hiseq2500–2 × 250 bp read length.

### 2.5 16S rRNA sequence data analysis

Sequence data was analyzed using Quantitative Insights into Microbial Ecology (QIIME v2) for the removal of primers and spacers from the sequences as described before (42, 43). Divisive Amplicon Denoising Algorithm 2 (DADA2) was utilized for trimming, denoising, merging paired-end forward and reverse reads, and removing the chimera sequences (44). OTU clustering was performed on denoised (DADA2) sequences using q2-vsearch tool in QIIME2. Forward and reverse reads were trimmed in case of low-quality reads (Q < 25%). Obtained feature table was rarefied using the diversity core-metrics-phylogenetic (q2-diversity) plugin in QIIME2 using a sample depth of 12,000, which was utilized to calculate microbial *α*- and *β*-diversity metrics. Abundance of microbes under difference taxa was elucidated by utilizing the reads in the feature table and reference taxonomic annotations from Silva database (release 138). Reads were extracted using 99% of 16S coverage and raw.taxonomy files that was trained using Naive Bayes classifier. The resultant trained classifier was then used with representative sequences produced from DADA2 for assigning sequences to individual taxa. 0.5% sequences of low abundance were filtered out.

### 2.6 Statistical analysis

All data were analyzed by one-way analysis of variance (ANOVA) followed by Tukey’s *post hoc* test and represented as mean ± S.E.M. using GraphPad Prism (V8). Data having unequal variances were log-transformed to achieve equal variances. Pearson correlation coefficients were calculated by linear regression to determine pair-wise associations between independent variables. Microbiome abundance data were represented as fraction of 1 (one) after low-count quality filtering of <5% and unclassified reads were filtered out. Data scaling was performed by total sum scaling and relative log expression was used for data transformation. The core microbiome profile was calculated based on >80% commonality across samples. To identify common co-regulatory networks at genus-level independent of treatments, a Debiased Sparse Partial Correlation (DSPC)-based correlative network was established (degree filter 2 and betweenness filter 1) as described previously (45), where nodes represented independent genera and edges represented the extent of associations. A false discovery rate (FDR)-adjusted q-value <0.2 was applied for the DSPC analysis. Partial least squares-discriminant analysis (sPLS-DA) was performed for dimension reduction. Variable Importance in Projection (VIP) scores were estimated for independent variables used in the PLS model.

## 3 Results

### 3.1 Interpersonal variation in baseline microbiome profile likely dictated by lifestyle

**Figure 2:**
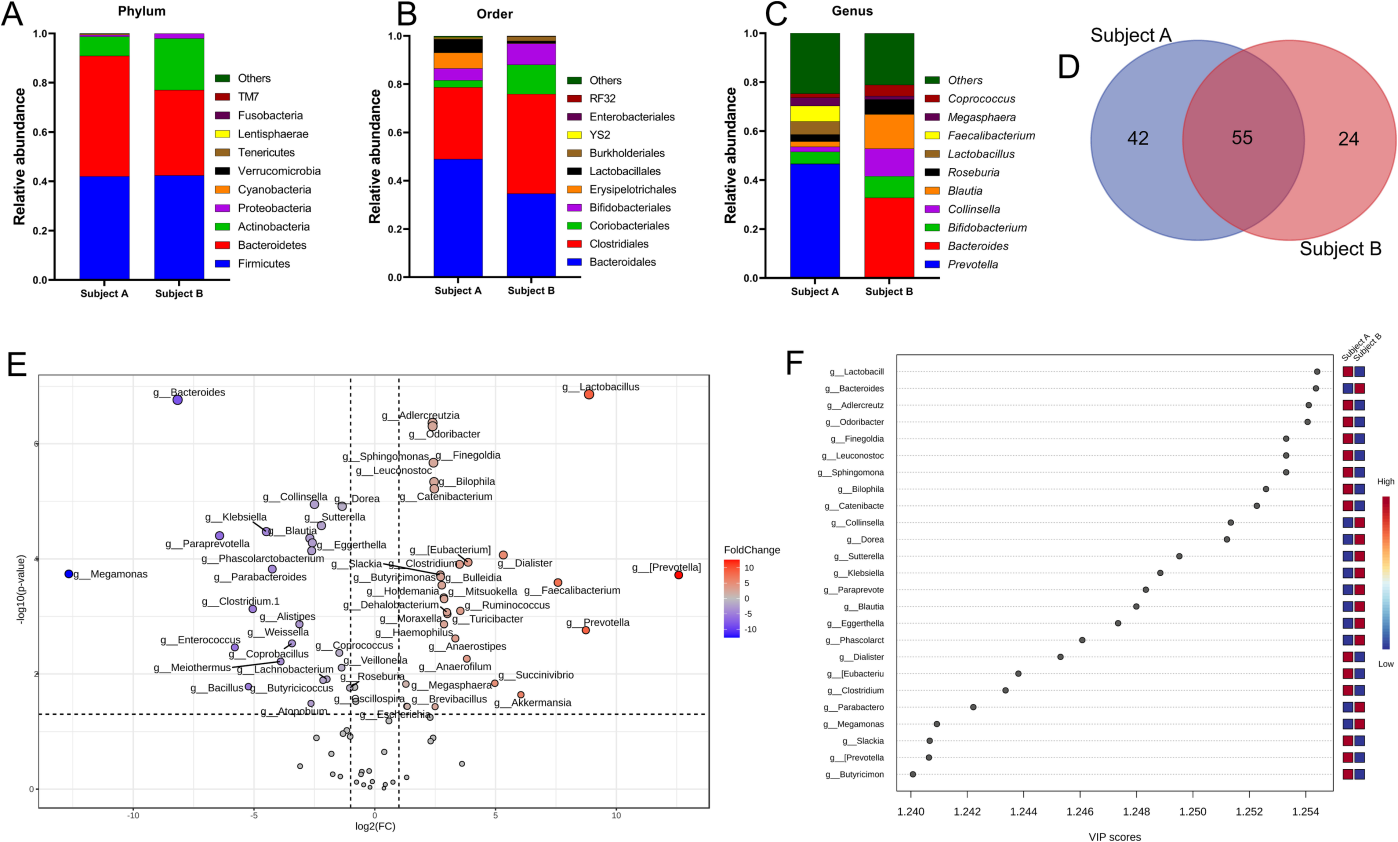
Inter personal gut microbiome profile at baseline. **(A–C)** Proportions of microbes at phylum, order and genus level. Taxa abundance were calculated as proportion of 1. **(D)** Venn diagram depicting common and unique set of microbes. **(E)** Volcano plot showing fold change (FC) analysis and T-tests (*p* < 0.05), highlighting relevant enriched microbial genera. Each point represents specific genera with its magnitude fold change (log2 of relative abundance) along the x-axis and level of significance (−log10 of *p* value) along the y-axis. The dotted line represents significance cutoff at *p* < 0.05. Points having magnitude of fold change <1 are represented in gray. **(F)** Result from Partial Least Squares Discriminant Analysis highlights variable importance in projection (VIP) and the weighted sum of absolute regression coefficients. The colored boxes indicate the relative abundance of corresponding genera in both subjects.

In the present study, the selected fecal matter donors differed in age, dietary habits and presence of chronic disease history (Figure 1B), factors that predominantly dictate the gut microbial phenotype (1, 46). Indeed, data showed significant inter-individual variance in the gut microbial profile at different taxonomic levels (Figures 2A–C). At the phylum level, compared to subject B, while Bacteroidetes was high in subject A, subject B had an increased abundance of Proteobacteria and Actinobacteria. At the order level, Bacteroidales, Lactobacillales, and Erysipelotrichales were enriched in subject A, whereas Clostridiales, Bifidobacteriales, and Coriobacteriales were high in subject B. Compared to subject B, predominant genera in subject A were *Faecalibacterium, Lactobacillus* and *Prevotella,* whereas *Bacteroides, Blautia, Coprococcus, Megasphaera* and *Collinsella* were high in subject B. Collectively, both individuals demonstrated distinct yet overlapping gut microbial patterns at the genus level (Figure 2D). Taxa distribution and variability comparing both individuals indicated *Lactobacillus*, *Bacteroides, Aldercreutzi, Odoribacter* and *Finegoldia* as the top candidates across the genus level having most significant fold change interpersonal difference (Figures 2E,F).

### 3.2 Probiotic, prebiotic and synbiotic differentially altered interpersonal gut microbiome

**Figure 3:**
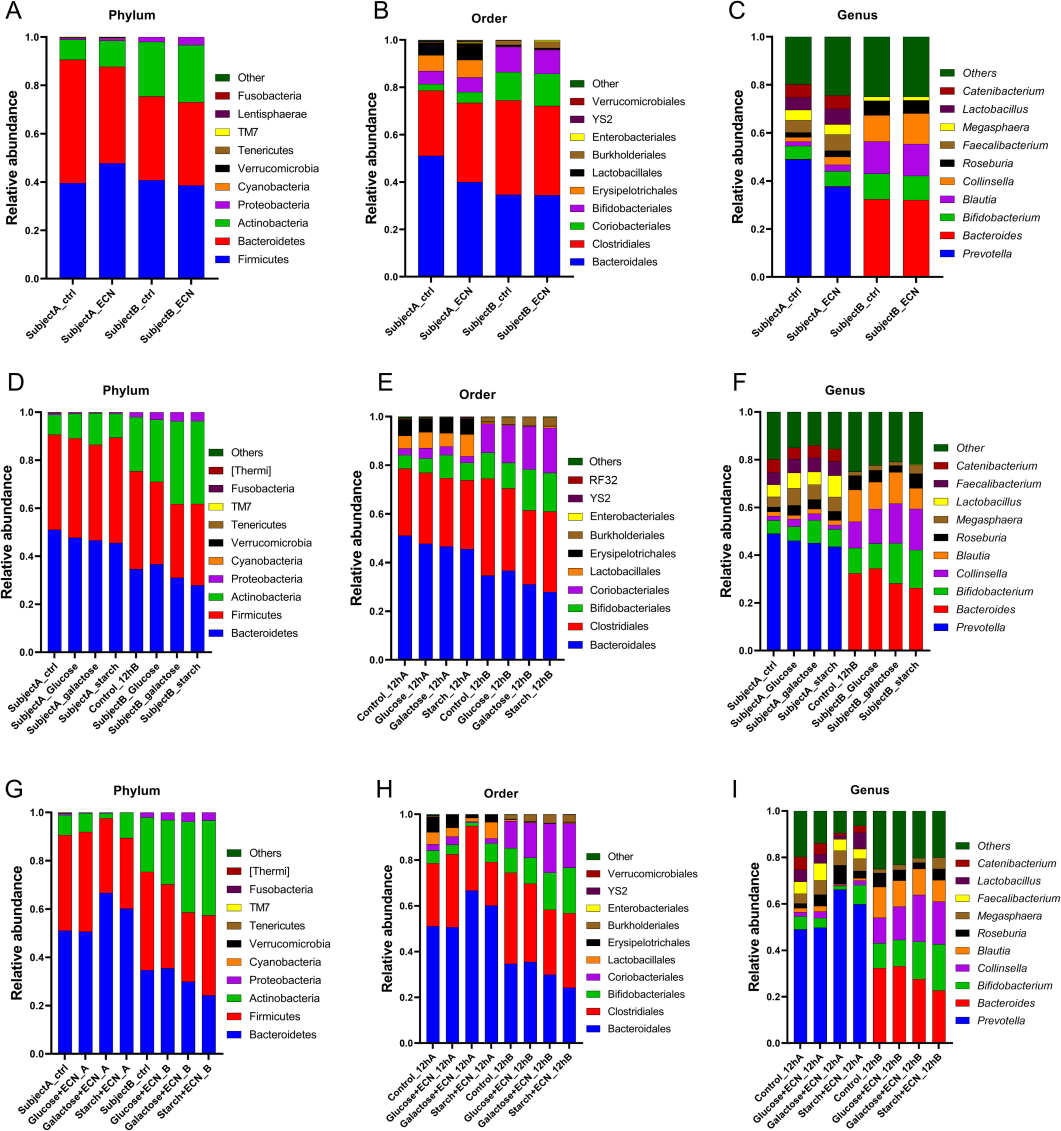
Inter personal microbiome variation at phylum, order and genus level under **(A–C)** probiotic, **(D–F)** prebiotic and **(G–I)** synbiotic treatment. Data represented as fraction of 1 after filtering unclassified reads. Abbreviation: ECN, *Escherichia coli* Nissle 1917.

To decipher the interpersonal microbiome variation under various treatments, taxonomic abundance patterns were segregated based on individual subjects (Figures 3A–I; Supplementary Tables 1–3). Data showed that for ECN treatment alone, top three phyla enrichments were observed in case of *Cyanobacteria* in subject B, and *Tenericutes* and *TM7* in subject A (Figure 3A). *Bacteroidetes* was decreased whereas *Firmicutes* was increased only in subject A. At order level, *Coriobacteriales* and *Verrucomicrobiales* were enriched only in subject A, whereas *Enterobacterales* was enriched in both *subjects* (Figure 3B). At the genus level, *Prevotella* was depleted, while predominant enrichment was observed for *Bacteroides, Blautia,* and *Collinsella* in subject A, and *Lactobacillus* in subject B. Taxa in subject B demonstrated a relatively stable profile under ECN treatment compared to subject A (Figure 3C).

In the case of prebiotic treatment (Figures 3D–F; Supplementary Table 2) at the phylum level, enrichment of *Actinobacteria* was evident in both subjects across all treatments (Figure 3D). *Proteobacteria* and *Cyanobacteria* were enriched in subject B but depleted in subject A. At the order level, Bifidobacteriales were elevated in both subjects for galactose and glucose treatment and Coriobacteriales under all treatments except for starch in subject A (Figure 3E). Lactobacillales was enriched under glucose and starch treatment of subject A, whereas Burkholderiales was elevated in subject B for all treatments. At the genus level, *Bifidobacterium* was elevated in both subjects other than under glucose treatments, and *Collinsella* was elevated across all groups except for starch in subject A (Figure 3F). Both *Roseburia* and *Megasphaera* were enriched in subject A, while only under glucose and starch treatment of subject A. At genus level, enrichment of *Bifidobacterium* was observed under galactose and starch treatment for both subjects, *Collinsella* except for starch treatment in subject A, *Roseburia* in all treatments of sample A, *Megasphaera* except for galactose in subject B, and *Lactobacillus* except for galactose in subject A and glucose in subject B was enriched altogether. *Bacteroides* was depleted in subject A. For subject B, *Blautia* was depleted under glucose and starch treatment, and, while *Roseburia* decreased under glucose and galactose treatment. Interestingly, *Faecalibacterium* was slightly enriched in subject A, whereas considerably depleted in subject B.

In case of synbiotic treatment (Figures 3G–I; Supplementary Table 3), *Proteobacteria* and *Cyanobacteria* were enriched for all treatments of subject B, but depleted under subject A (Figure 3G). At the order level, *Coriobacteriales*, *Burkholderiales*, and *Enterobacteriales* were enriched in all treatments of subject B (Figure 3H). At the genus level, *Roseburia* and *Megasphaera* were enriched in all treatments of subject A, while *Collinsella, Blautia,* and *Faecalibacterium* were only enriched under the glucose + ECN treatment of subject A (Figure 3I). Similarly, *Bifidobacterium* and *Lactobacillus* were enriched under starch + ECN treatment of subject A. In the case of subject B, *Bifidobacterium* and *Collinsella* were elevated under both galactose + ECN and starch+ECN treatments; however, *Megasphaera* and *Lactobacillus* were elevated only under starch + ECN treatment. *Blautia* was depleted in all treatments except for glucose+ECN in subject A, and *Bacteroides* was reduced in all synbiotic treatments of subject A.

### 3.3 Overall interpersonal microbial patterns under treatments

**Figure 4:**
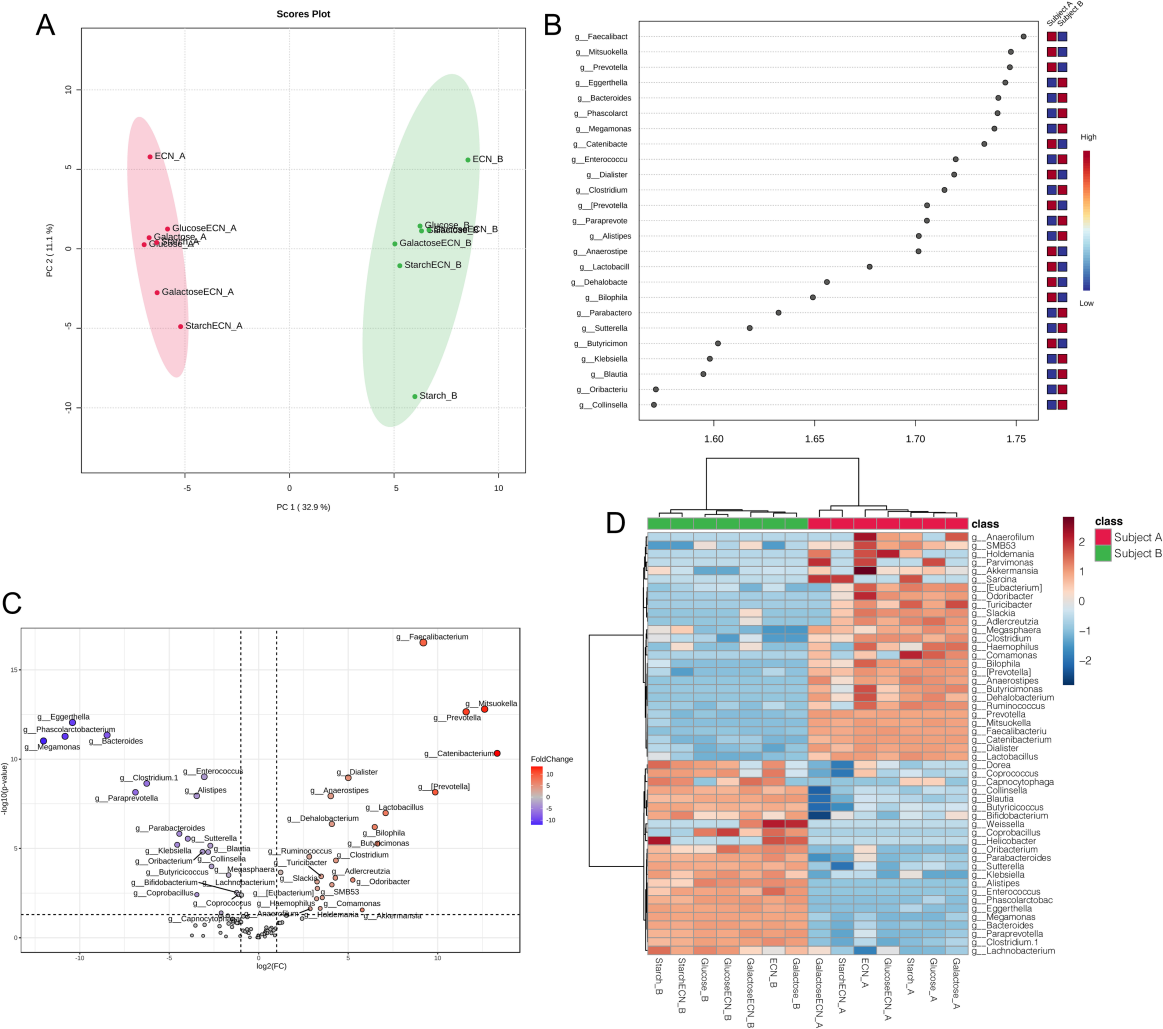
Gut microbial patterns associated with inter personal microbiome variation under various treatments. **(A)** Partial Least Squares Discriminant Analysis (PLSDA) for gut microbial patterns of fecal slurries of both subjects under probiotic, prebiotic and synbiotic treatments. **(B)** Result from PLSDA highlights variable importance in projection (VIP) and the weighted sum of absolute regression coefficients. The colored boxes indicate the relative abundance of corresponding genera in the fecal matter of both subjects under various treatments. **(C)** Volcano plot showing fold change (FC) analysis and T-tests (*p* < 0.05), highlighting relevant enriched microbial genera between both subjects under various treatments. Each point represents specific genera with its magnitude fold change (log2 of relative abundance) along the x-axis and level of significance (−log10 of p value) along the y-axis. The dotted line represents significance cutoff at *p* < 0.05. Points having magnitude of fold change <1 are represented in gray. **(D)** Correlation between top 50 genera (based on abundance) with treatment effects on the fecal slurries of both subjects. Abbreviation: ECN, *Escherichia coli* Nissle 1917.

Multivariate analysis based on PLSDA clearly segregated all treatment effects between subject A and B, indicating distinct modulatory patterns of microbial genera in two individuals (Figure 4A). VIP scores plot summarized genera responsible for interpersonal microbial variability across all treatments that included *Faecalibacterium*, *Mitsuokella*, *Eggerthella, Prevotella,* and *Bacteroides* as the top five genera contributing to the variability (Figure 4B). Fold change depiction using volcano plot showed top 3 significant genera in subject A as *Faecalibacterium, Mitsuokella* and *Prevotella*, whereas the same was identified in subject B as *Eggerthella, Phascolarctobacterium* and *Bacteroides* (Figure 4C). Correlation heatmap indicated strong positive correlation of ECN_A with *Akkermansia, Anaerofilum,* and *Odoribacter*, while ECN_B has positively correlated with *Weissella* (Figure 4D). *Parvimonas* was positively correlated with galactose+ECN, ECN, and Glucose treatment in subject A, *Sarcina* with galactose+ECN, starch+ECN and starch treatment, and *Comamonas* with starch treatment in subject A. *Collinsella, Blautia, Butyricoccus,* and *Bifidobacterium* were inversely correlated with galactose+ECN treatment in subject A. In case of subject A, *Weissella* was positively correlated with ECN and galactose treatment, whereas *Helicobacter* with starch treatment.

### 3.4 Probiotic, prebiotic, and synbiotic differentially altered gut microbial abundance independent of diversity

**Figure 5:**
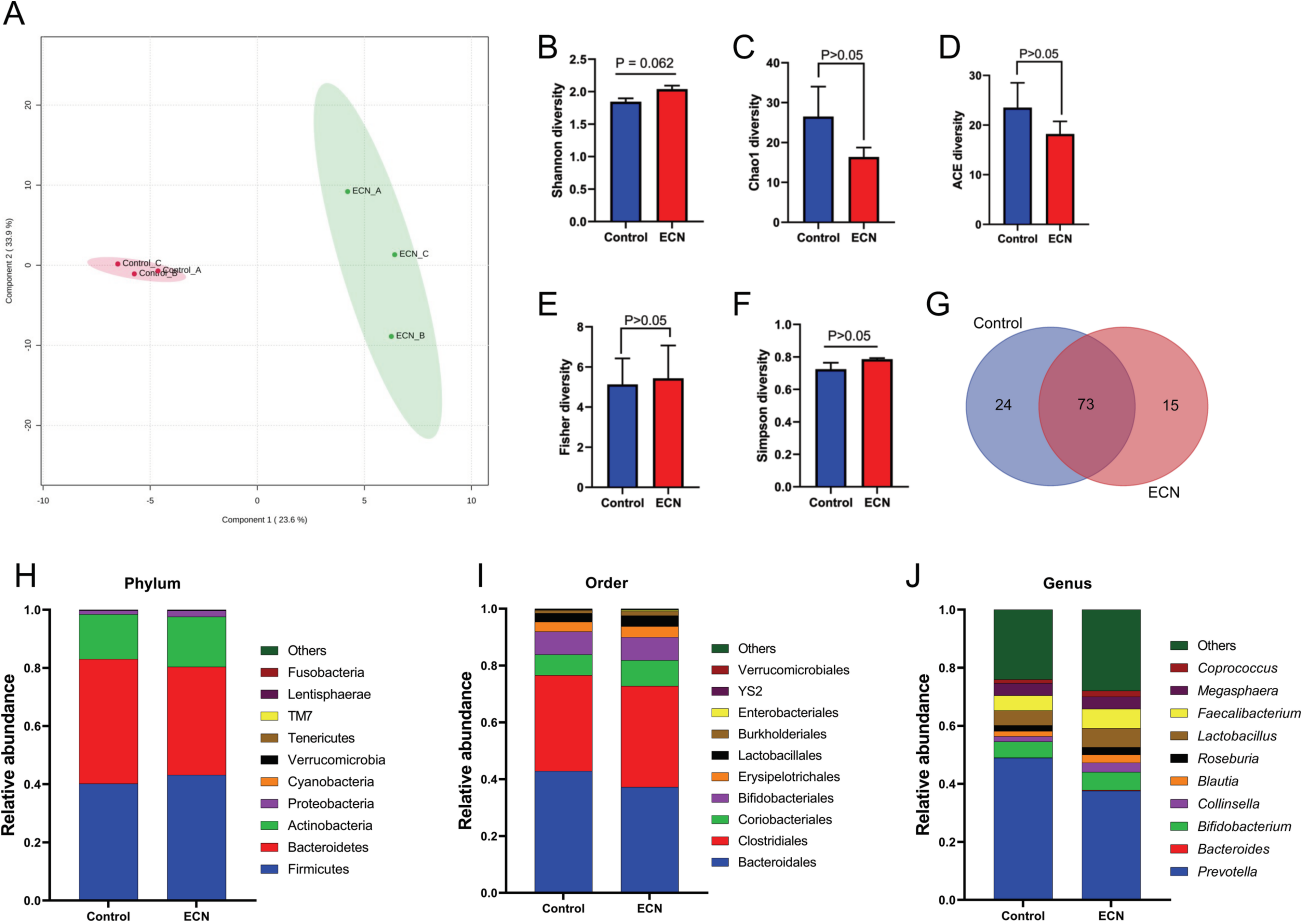
Effect of probiotic (ECN) on gut microbiota. **(A)** Partial least squares-discriminant analysis (PLSDA) reveals discriminating characteristics of microbiota segregating untreated control from that of ECN-treatment for *β*-diversity. **(B–F)** Various gut microbial *α*-diversity metrics indicating variability in microbial community composition. **(G)** Venn diagram depicting commonality and uniqueness of identified genera between control and ECN. **(H–J)** Taxa abundance at phylum, order and genus level represented as proportions of 1.

**Figure 6:**
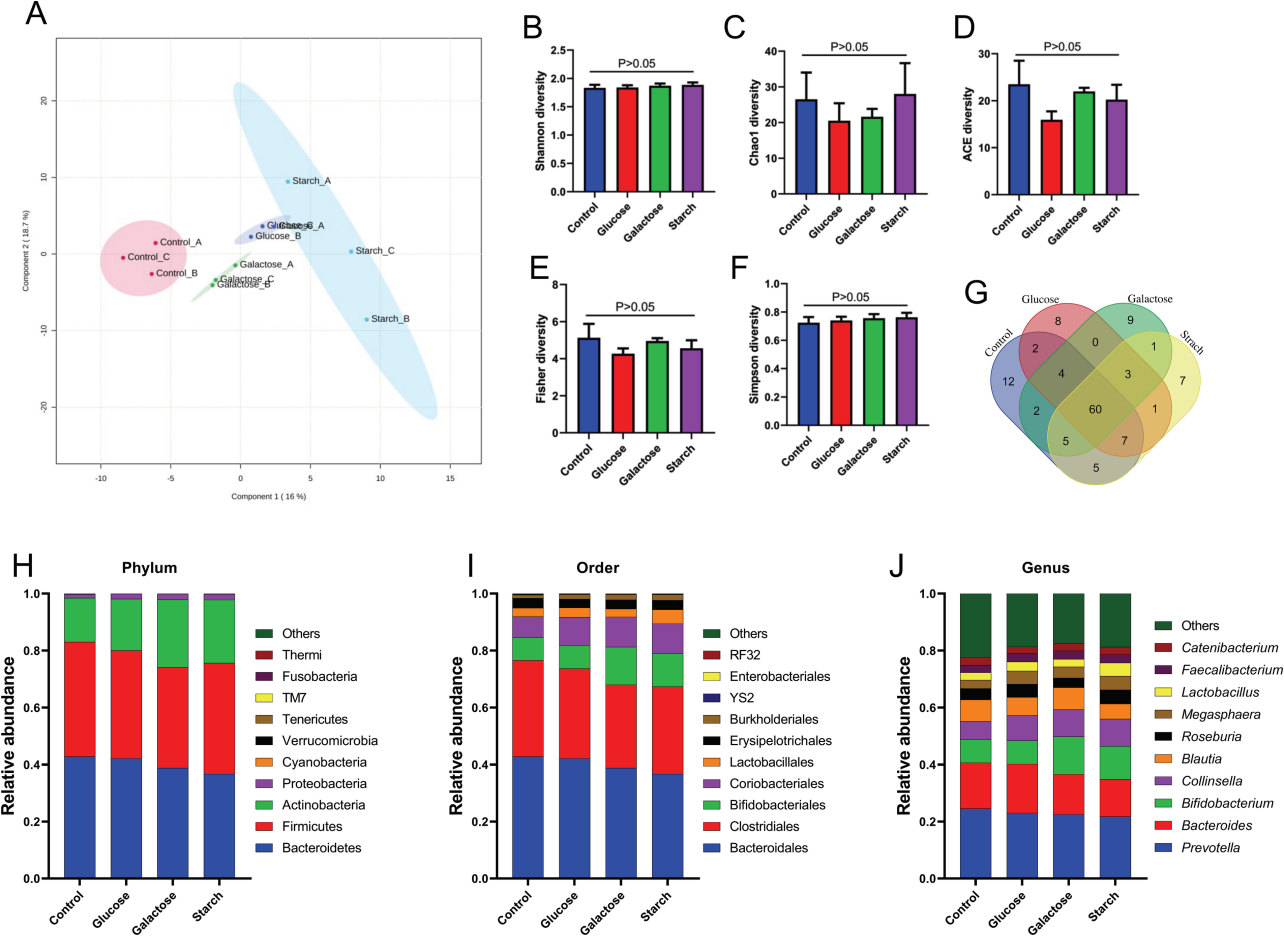
Effect of prebiotic (glucose, galactose and starch) on gut microbiota. **(A)** Partial least squares-discriminant analysis (PLSDA) reveals discriminating characteristics of microbiota segregating groups based on β-diversity. **(B–F)** Various gut microbial α-diversity metrics indicating variability in microbial community composition. **(G)** Venn diagram depicting commonality and uniqueness of identified genera between groups. **(H–J)** Abundance of taxa at phylum, order and genus level represented as proportions of 1.

**Figure 7:**
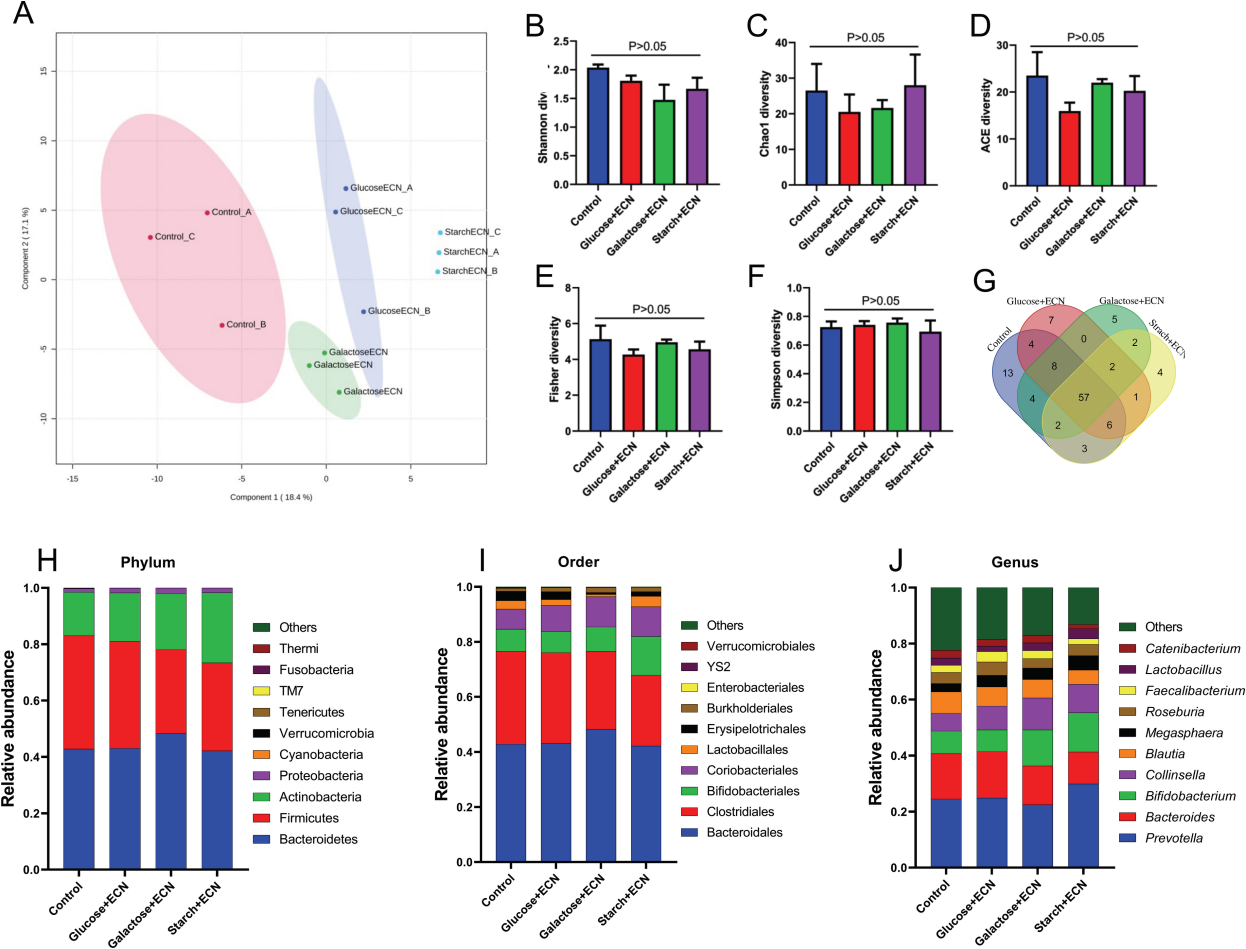
Effect of synbiotics (ECN in combination with either glucose, galactose or starch) on gut microbiota. **(A)** Partial least squares-discriminant analysis (PLSDA) reveals discriminating characteristics of microbiota segregating groups based on β-diversity. **(B–F)** Various gut microbial α-diversity metrics indicating variability in microbial community composition. **(G)** Venn diagram depicting commonality and uniqueness of identified genera between groups. **(H–J)** Abundance of taxa at phylum, order and genus level represented as proportions of 1.

We intended to understand the independent effects of probiotic, prebiotic, and synbiotic treatments on the gut microbiota (Figures 5–7; Supplementary Figure 1). For this purpose, the sequence data were analyzed based on overall treatment effects and compared with untreated controls. Data showed clear segregation among all treatments at the *β*-diversity level, depicting dissimilarity between microbial communities (Figures 5A, 6A, 7A). Nevertheless, for *α*-diversity no difference was observed (Figures 5B–F, 6B-F, 7B–F), likely due to low statistical power. However, separate α-diversity comparison of prebiotics and synbiotics demonstrated a significant difference in OTU number (ACE diversity) between galactose and glucose+ECN and starch+ECN, starch, and starch+ECN (Supplementary Figure 1D). In case of taxa evenness (Fisher diversity), difference was observed only between galactose and starch+ECN (Supplementary Figure 1E). Venn diagram at genus level showed that majority of the taxa were commonly shared by all groups (Figures 5, 6, 7G). However, an abundance of taxa at various phylogenetic levels differed among the treatments (Figures 5, 6, 7H–J). For instance, compared to the control, ECN treatment highly elevated the abundance of *Proteobacteria, Verrucomicrobia, Tenericutes,* and *TM7*. *Bacteroides, Bifidobacterium, Collinsella,* and *Coprococcus* were highly enriched, while *Prevotella* was depleted due to ECN treatment at the genus level (Figure 5J). *Collinsella* and *Megasphaera* were enriched due to all prebiotic treatments, while *Bifidobacterium* was enriched only due to glucose and starch treatment and *Lactobacillus* was enriched under glucose and starch treatment (Figure 6J). Under synbiotic treatment, *Blautia* and *Catenibacterium* were depleted, while *Collinsella* and *Megasphaera* were enriched under all treatment (Figure 7J). The abundance of *Prevotella* only under starch+ECN, *Bifidobacterium* under galactose+ECN and starch+ECN, *Faecalibacterium* under glucose+ECN, and *Lactobacillus* only under starch+ECN were elevated. Comparison between probiotic and corresponding synbiotic treatment (Supplementary Table 7) revealed enrichment of *Bacteroides, Bifidobacterium, Blautia, Collinsella,* and *Roseburia* and depletion of *Faecalibacterium, Lactobacillus,* and *Prevotella* due to synbiotic treatment. Moreover, a comparison between prebiotic and corresponding synbiotic showed a relatively stable gut microbial pattern other than an increase of *Bifidobacterium* and *Prevotella* under starch+ECN, and *Faecalibacterium* under galactose+ECN, while *Catenibacterium* was depleted under starch+ECN treatment (Supplementary Table 8).

### 3.5 Limited treatment effects on core microbiome profile

Next, we identify variations in the core microbiome profile associated with treatment effects (Supplementary Figures 2–4). Data showed no difference in the core microbiome due to ECN treatment, although a difference in abundance was observed. Samples under prebiotic treatment had *Bifidobacterium, Collinsella, Blautia, Roseburia,* and *Megasphaera* common as part of the core microbiome. Samples treated with galactose and starch had *Coprococcus* as an additional core microbiome. In the case of synbiotic treatment*, Roseburia, Bifidobacterium,* and *Megasphaera* were common across treatments. However*, Blautia* was not identified as core microbiome under galactose treatment. The low variation in the core microbiome profile was likely attributed to the small sample size.

### 3.6 Modulation of distinct microbes likely responsible for probiotic, prebiotic and synbiotic effects

**Figure 8:**
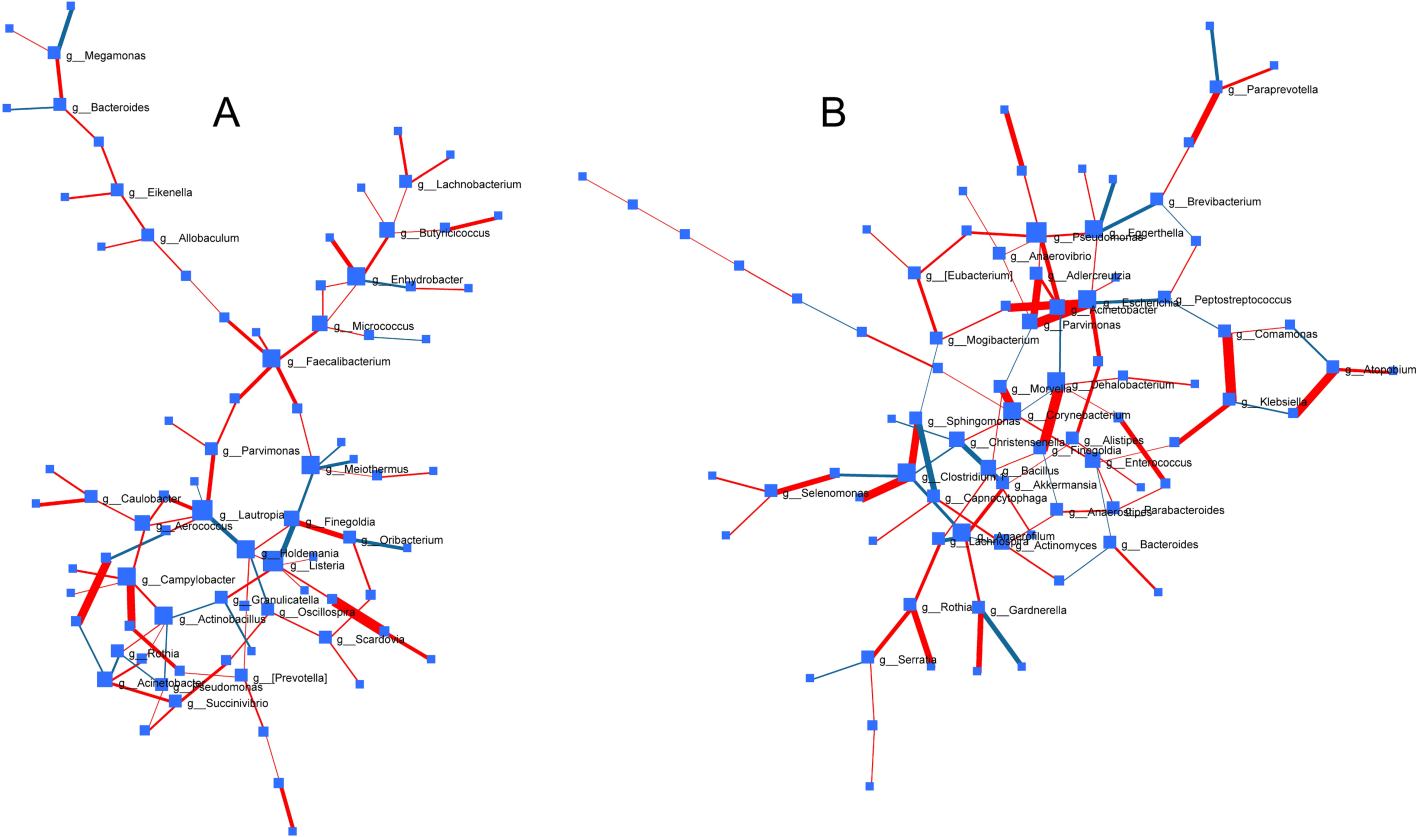
Correlation network of bacterial genera across treatments based on Debiased Sparse Partial Correlation (DSPC) analysis depicting pattern of gut microbial associations under the influence of **(A)** prebiotics and **(B)** synbiotics. Size of nodes represents direction of change and thickness of edges indicates extent of significance. Red edge and blue edge represented positive and negative correlation, respectively. No significant correlation was observed in case of probiotic treatment under common degree and betweenness filter.

To decipher common microbes impacted and co-regulated under the influence of probiotic, prebiotic and synbiotic treatments, DSPC-based correlation analysis was performed (Figure 8). While no common significant patterns (*p* > 0.05) were identified under ECN treatment, prebiotic treatment revealed strong positive correlation between *Prevotella* and *Selenomonas* (*p* = 0.006, *r* = 1) followed by *Porphyromonas and Leptotrichia* (*p* = 0.013, *r* = 0.86), *Caulobacter* and *Lactobacillus* (*p* = 0.015, *r* = 0.91), *Finegoldia* and *Oribacterium* (*p* = 0.043, *r* = 0.67). Inverse association was only identified between *Finegoldia* and *Listeria* (*p* = 0.045, *r* = −0.68). In case of synbiotics, positive correlations were observed between *Comamonas* and *Klebsiella* (*p* = 0.0104, *r* = 0.91), *Dehalobacterium* and *Finegoldia* (*p* = 0.0107, *r* = 0.959), *Parvimonas* and *Escherichia* (*p* = 0.0147, *r* = 0.944), *Atopobium* and *Neisseria* (*p* = 0.0194, *r* = 0.91), *Actinobacillus* and *Clostridium-1* (*p* = 0.0194, *r* = 0.91*), Lactobacillus and Escherichia* (*p* = 0.0197, *r* = 0.899), *Adlercreutzia* and *Parvimonas* (*p* = 0.0255, *r* = 0.854), *Sphingomonas and Clostridium* 1 (*p* = 0.0255, *r* = 0.854), *Paraprevotella* and *WAL_1855D* (*p* = 0.0348, *r* = 0.723), *Gardnerella* and *Butyricimonas* (*p* = 0.0468, *r* = 0.691), *Streptococcus and Selenomonas* (*p* = 0.0475, *r* = 0.674), *Rothia and Porphyromonas* (*p* = 0.04, *r* = 0.749). Inverse correlation was observed only between *Sphingomonas* and *Capnocytophaga* (*p* = 0.041, *r* = −0.68).

## 4 Discussion

This research aimed to highlight the intricate relationships between interpersonal differences in gut microbial composition and their response to probiotic therapies. Our primary hypothesis was that variations in the makeup of gut microbiota substantially impact the degree and nature of microbial changes caused by probiotics. To investigate this, we performed a comparative study of the changes in gut microbiome when exposed to probiotics, prebiotics, and synbiotics. We used a host-free model system to create a controlled environment that is not influenced by host-specific factors. This resulted in lower intra- and inter-group variability that is generally observed in metagenomic studies. Additionally, we examined the effects of structural variations of microbially digestible sugars (i.e., glucose, galactose and starch), on the gut microbial communities. Two structurally similar monosaccharides (i.e., glucose and galactose differing only at C4 -OH group orientation) were compared with oligosaccharide for their potentials to modulate the gut microbiome. Earlier study showed comparatively rapid metabolism of glucose relative to galactose by human oral microbiome (47). Although galactose could be microbially converted to glucose-6-phosphate through the Leloir pathway for energy production and as carbon source, utilization of glucose is more efficient than any other types of sugar (48). Under host-free system that is devoid of amylase, starch digestion is a multistep process incorporating multiple bacterial species cooperating to metabolize starch for obtaining glucose (49).

The notable variations in the gut microbial profiles of the two individuals highlight the substantial influence of food, lifestyle, health condition, and age on the composition of microbiota. The observed disparities in microbial makeup are likely attributable to the unique dietary patterns and lifestyle behaviors of the two individuals as indicated before (50–52). Subject A’s elevated levels of *Prevotella* and *Lactobacillus* are likely triggered by intake of high fiber and low-western-type diet, whereas increased abundance of *Bacteroides* and *Blautia* are linked to low fiber and high western-type diet (53, 54). Consuming diets rich in fruits, vegetables, and whole grains promotes the growth of commensal bacteria by providing favorable nutrients. The significant prevalence of *Proteobacteria* and *Actinobacteria* in the gut of Subject B suggests a dietary pattern similar to a Western diet that is low in fiber but rich in refined sugar, likely leading to a risk of non-communicable disease in the future (55). Similar to our data, others have shown that an Indian-type non-vegetarian diet increases *Prevotella*, as observed in subject A (56). Lifestyle variables such as levels of physical activity, stress, smoking, and alcohol use consistently impact microbial populations. The microbiota of Subject A, characterized by elevated *Erysipelotrichales* was linked to alcohol consumption (57), while elevated *Coriobacteriales* in subject B was linked with habitual smoking (58). Health problems, such as metabolic disorders, gastrointestinal diseases and aging, significantly impact the composition of gut microbial communities. The microbiota of Subject A, which had higher numbers of potentially harmful genera like *Proteobacteria* and *Erysipelotrichaceae*, could be associated with aging and presence of chronic conditions like metabolic syndrome, obesity, or inflammatory diseases (59). Finally, the fact that gut microbial patterns are strongly impacted by diet and lifestyle, but are not universal, is well established (1, 6). In line, high interpersonal variability in the abundance several minor genera such as *Aldercreutzia*, *Odoribacter*, *Finegoldia*, and others were observed. Although, strong association of these bacteria with health and lifestyle status remains underexplored, but these minor phyla has been predicted as key players in the maintenance of the gut microbial eubiosis (60).

Prior study utilizing supplementation of probiotic *Bifidobacterium infantis* indicate no apparent changes in the gut microbial composition and diversity in healthy adults (61). In line with these observations, although our data show no dramatic change in the overall microbial *α*-diversity including that of core microbiome profile, *Prevotella* was depleted and *Faecalibacterium* was enriched due to ECN treatment. This was supported by others demonstrating that ECN modulates the population of *Prevotella* and *Faecalibacterium* in a similar trend (62). At the interpersonal microbial variation, ECN-induced changes were relatively stable across all taxonomic levels in subject B. The enrichment of *Bifidobacterium* under galactose and starch treatment in both subjects was supported by the fact that *Bifidobacterium* can metabolize sugar through alternative “bifid shunt” pathway (63), but the key enzyme fructose-6-phosphate phosphoketolase could be inhibited in the presence of other members of the microbiome (64). Similarly, the enrichment of *Megasphaera* and *Collinsella* in prebiotic treatment of subjects A and B, respectively (except for starch in subject A and galactose in subject B), are likely dictated by the presence or absence of other microbial genera in the community. Indeed, a plethora of disease-associated data shows that survival and overgrowth of certain commensals could be associated with mutualistic interaction with other bacterial species within the community that provides desired nutrients and facilitate colonization success to the commensals (65, 66). The enrichment of butyrate-producing *Roseburia* only in subject A due to prebiotic treatment could be associated with increased dietary intake of fibers by subject A (54).

*Megasphaera* in subject A was enriched in all synbiotic and prebiotic treatments but depleted due to ECN, indicating the fact that the prebiotic composition is likely superior to ECN in elevating the abundance of *Megasphaera*. A similar trend was observed in the case of *Bifidobacterium*, which was primarily enriched due to synbiotics containing galactose and starch; its abundance was marginally enriched due to ECN but significantly higher when treated with galactose and starch alone. This indicated that the bloom on *Bifidobacterium* under synbiotics were governed by the prebiotic component of the formulation. Indeed, it was shown by others that oral ECN treatment in patients with hepatic encephalopathy do not increase the abundance of *Bifidobacterium* (67) whereas prebiotic diet could lead to Bifidogenic effects in patients with non-alcoholic fatty liver disease (68). Interestingly, *Collinsella*, which covered a large proportion of the total genera in subject B, was highly enriched due to both prebiotic and synbiotic treatment, but marginally due to ECN. This was observed despite the fact that *Collinsella* sp. has been postulated as a predictive marker of response to probiotic treatment both in subjects with irritable bowel disease (69) and in healthy individuals (70). *Prevotella* was predominant only in subject A and was further enriched only due to synbiotics containing galactose and starch, but not due to ECN or prebiotics alone.

Our data showed that, similar to microbial taxa diversity, ECN treatment had no apparent impact on the core microbiome profile. However, limited impact was recorded under prebiotic and synbiotic treatment. This remains consistent with earlier pre-clinical and clinical studies demonstrating that the core microbiome is a relatively stable phenotype and is highly resistant to acute treatment (51, 71, 72). Indeed, prior studies show that probiotic *Lactobacillus* strains isolated from fermented milk do not alter the core microbiome and microbial diversity in gnotobiotic mice and in monozygotic twins (73). However, in our study, prebiotics alone or as part of the synbiotic formulation are expected to have a greater impact on the core microbiome since, under a host-free system, the added sugars serve as a carbon source for the major taxa and subsequently impact their abundance. Indeed, our data show only minor differences in the core microbiome profile and associated abundance under both prebiotic and synbiotic treatment.

Our data clearly demonstrated differential impacts of probiotic, prebiotic and synbiotic treatment that were closely associated with the baseline inter-individual microbiome profiles. Nevertheless, due to innate variability in the biological system impacted by lifestyle and genetic factors, the inter-individual microbiome phenotype could be influenced (74), as seen in the current study with individuals with varying lifestyles and health statuses. Indeed, others have shown that impacted by lifestyle; interpersonal differences contribute to variation in microbiota composition more than that of other physiological variables like hormonal levels (75). Thus, microbial patterns commonly influenced by the treatment effects were identified using correlation networks demonstrating related impacts on paired microbial genera. This was additionally expected to reflect the role of the minor phyla within the fecal microbial community, often termed as the ‘dark matter’ (76, 77). Interestingly, in line with the negligible impact of ECN on the inter-individual microbial phenotype (except for the depletion of *Prevotella* in subject A), no coregulatory networks were identified under ECN treatment. Under prebiotic treatment, a strong association between *Prevotella* and *Selenomonas* was likely supported by the similarities in their carbohydrate metabolizing enzymatic machinery facilitating metabolic crossfeeding (78). Indeed, comparatively recent studies indicate interspecies cross-feeding, including that of *Prevotella* and *Selenomonas*, for the degradation of a variety of carbohydrates as carbon sources (79). The close positive association observed between *Lactobacillus* and *Escherichia* under synbiotic treatment is likely due to the supplementation of ECN as part of synbiotic formulation and prebiotic effects of the sugars within the same formulation. Indeed, using various carbohydrate sources, it was demonstrated that the growth and metabolic activities of various *Lactobacillus* sp. are dependent on the carbohydrate source (80), similar to our data where the abundance of *Lactobacillus* was differentially impacted based on type of carbohydrate. The close positive association between *Adlercreutzia* and *Parvimonas* under synbiotic treatment was supported by the fact that presence of dietary fiber can modulate the abundance of both genera to similar extent in healthy adults (81). Interestingly, positive association between several minor genera (e.g., *Finegoldia, Porphyromonas, Leptotrichia, Listeria*, etc.) were observed under the influence of both prebiotic and synbiotic treatment. Majority of these commensal genera are known to cause opportunistic infections and could be regulated by prebiotic and probiotic treatment (5, 65).

Finally, it is to be acknowledged that the current study possesses certain limitations. One of them is small cohort size which although provided definite outcomes based on inter-personal microbiome variations, but was unable to provide a broader picture based on lifestyle factors. This also likely resulted in loss of statistical power for certain analysis (*α*-diversity). Nevertheless, a small sample size with defined microbiome profile facilitated ease in data interpretation under related prebiotic, probiotic and synbiotic treatments, that would likely be challenging using larger cohort, which would introduce undefined variability. Another limitation is lack of predicted microbial metabolic data that is generally computed based on 16 S sequences, that also limited interpretation of the microbial correlation network data. Nevertheless, functional data are considered valid based on functional assessment either based on culturomics strategies or gene expression studies of specific enriched pathways, which were out of scope of the current study.

## 5 Conclusion

Our findings highlight the significant influence that heterogeneity in interpersonal microbiomes has on the effectiveness of individualized diet and treatment plans. Using a regulated host-free model system, we showed that ECN-based probiotic, prebiotic and synbiotic treatment may not be universally effective and that baseline microbiome composition strongly affects treatment results. The research participants’ distinct microbial profiles were influenced by their pre-existing health issues, eating habits, and lifestyle choices. These factors also determined how the patients responded to different therapies. Importantly, probiotic ECN therapy had limited impact, indicating that a one-size-fits-all strategy would not be successful, even when prebiotic and synbiotic therapies led to the enrichment of beneficial bacterial taxa including *Bifidobacterium* and *Lactobacillus*. Additionally, the study identified certain microbial relationships that are impacted by treatments. For example, the study highlighted the complex interactions among microbial communities by revealing a positive link between *Prevotella* and *Selenomonas* under prebiotic therapy. Collectively, the present study advocates that customized diet and treatment plans should be developed based on individual microbiome, taking into consideration its distinct makeup and the several variables that affect it. With its potential to maximize the effectiveness of dietary and pharmacological therapies, this customized strategy might lead to better gut health and overall well-being.

## Data Availability

The datasets presented in this study can be found in online repositories. The names of the repository/repositories and accession number(s) can be found in the article/Supplementary material.
