# Supplementary material for: Modulation of gut microbiome in response to the combination of Escherichia coli Nissle 1917 and sugars: a pilot study using host-free system reflecting impact on interpersonal microbiome
Source: Front Nutr. 2024 Oct 22;11:1452784. doi: 10.3389/fnut.2024.1452784 (PMC11534610; doi:10.3389/fnut.2024.1452784)
Supplement: Supplementary file 1 [file Data_Sheet_1.docx]

Supplementary Material

Modulation of Gut Microbiome in Response to the Combination of Escherichia Coli Nissle 1917 and Sugars: A Pilot Study Using Host-Free System Reflecting Impact on Interpersonal Microbiome

Kiran Heer^1,2 $^, Manpreet Kaur^1,$^, Dwinder Sidhu^3^, Priyankar Dey^3^* and Saumya Raychaudhuri^1^*

^1^Molecular Biology and Microbial Physiology Division, CSIR-Institute of Microbial Technology, Chandigarh, India

^2^Academy of Scientific and Innovative Research (AcSIR), Ghaziabad- 201002, India.

^3^Department of Biotechnology, Thapar Institute of Engineering & Technology, Patiala, Punjab, India

$: Equal Authorship

* Correspondence**:**Saumya Raychaudhuri: saumya@rocketmail.com, saumya@imtech.res.in

Priyankar Dey: priyankar.dey@thapar.edu

Keywords: Escherichia coli Nissle 1917; Probiotic; Prebiotic; Synbiotic; Microbiome; Inter-individual variation

**Supplementary Table 1.** Represents the extent of times change in microbial taxa in two different subjects due to probiotic treatement. Data corresponds to Fig. 3A-C.

|  | **Subject A** | **Subject B** |
| --- | --- | --- |
|  | **ECN** | **ECN** |
|  | **Phylum** | |
| Firmicutes | 1.21 | 0.95 |
| Bacteroidetes | 0.78 | 0.99 |
| Actinobacteria | 1.3 | 1.05 |
| Proteobacteria | 1.34 | 1.64 |
| Cyanobacteria | 0.99 | 6.14 |
| Verrucomicrobia | 1.89 |  |
| Tenericutes | 2.2 |  |
| TM7 | 2.41 | 0 |
| Lentisphaerae | 0.88 |  |
| Fusobacteria |  | 0.92 |
| Other | 1.65 | 1.23 |
|  | **Order** | |
| Bacteroidales | 0.78 | 0.99 |
| Clostridiales | 1.21 | 0.95 |
| Coriobacteriales | 1.68 | 1.14 |
| Bifidobacteriales | 1.12 | 0.94 |
| Erysipelotrichales | 1.11 | 1.09 |
| Lactobacillales | 1.3 | 1.02 |
| Burkholderiales | 1.05 | 1.43 |
| Enterobacteriales | 2.33 | 5.23 |
| YS2 | 0.99 |  |
| Verrucomicrobiales | 1.89 |  |
| Other | 1.36 | 0.98 |
|  | **Genus** | |
| Prevotella | 0.77 | 0.05 |
| Bacteroides | 1.64 | 1 |
| Bifidobacterium | 1.12 | 0.94 |
| Blautia | 1.47 | 0.99 |
| Collinsella | 1.85 | 1.16 |
| Roseburia | 1.33 | 0.9 |
| Faecalibacterium | 1.29 | 0.15 |
| Megasphaera | 0.99 | 0.92 |
| Lactobacillus | 1.29 | 1.46 |
| Catenibacterium | 1.01 |  |
| Others | 1.23 | 1 |

**Supplementary Table 2.** Represents the extent of times change in microbial taxa in two different subjects due to prebiotic treatement. Data corresponds to Fig. 3D-F.

|  | **Subject A** | | | **Subject B** | | |
| --- | --- | --- | --- | --- | --- | --- |
| **Phylum** | | | | | | |
|  | **Glucose** | **Galactose** | **Starch** | **Glucose** | **Galactose** | **Starch** |
| Bacteroidetes | 0.93 | 0.91 | 0.89 | 1.06 | 0.9 | 0.81 |
| Firmicutes | 1.05 | 1.01 | 1.11 | 0.84 | 0.75 | 0.83 |
| Actinobacteria | 1.23 | 1.58 | 1.19 | 1.15 | 1.53 | 1.53 |
| Proteobacteria | 0.76 | 0.47 | 0.67 | 1.53 | 1.84 | 1.82 |
| Cyanobacteria | 0.7 | 0.65 | 0.88 | 18.04 | 6.6 | 14.09 |
| Verrucomicrobia | 0.03 | 0.01 | 0.02 |  |  |  |
| Tenericutes | 0.3 | 0.52 | 1.46 |  |  |  |
| TM7 | 1.57 | 2.9 | 2.55 | 1 | 0 | 1.88 |
| Fusobacteria |  |  |  | 0.25 | 0 | 0.47 |
| [Thermi] |  |  |  | 0.29 | 0.16 | 0.13 |
| Others | 0.24 | 0.47 | 0.16 | 1 | 1.54 | 3.01 |
| **Order** | | | | | | |
| Bacteroidales | 0.93 | 0.91 | 0.89 | 1.06 | 0.9 | 0.81 |
| Clostridiales | 1.06 | 1.02 | 1.03 | 0.85 | 0.76 | 0.83 |
| Bifidobacteriales | 1.06 | 1.72 | 1.3 | 0.98 | 1.58 | 1.5 |
| Coriobacteriales | 1.58 | 1.3 | 0.97 | 1.3 | 1.49 | 1.55 |
| Lactobacillales | 1.22 | 1.04 | 1.71 | 0.38 | 0.31 | 1.04 |
| Erysipelotrichales | 0.86 | 0.93 | 0.97 | 1.05 | 0.41 | 0.81 |
| Burkholderiales | 0.92 | 0.51 | 0.74 | 1.59 | 1.93 | 1.91 |
| Enterobacteriales | 0.61 | 0.3 | 0.62 | 0.74 | 0.71 | 0.6 |
| YS2 | 0.7 | 0.65 | 0.88 |  |  |  |
| RF32 | 0.49 | 0.42 | 0.74 |  |  |  |
| Others | 0.29 | 0.36 | 0.4 | 0.6 | 0.39 | 1.62 |
| **Genus** | | | | | | |
| Prevotella | 0.94 | 0.92 | 0.89 | 0.07 | 0.09 | 0.2 |
| Bacteroides | 0.5 | 0.49 | 0.67 | 1.07 | 0.87 | 0.81 |
| Bifidobacterium | 1.06 | 1.72 | 1.3 | 0.98 | 1.58 | 1.5 |
| Collinsella | 1.82 | 1.56 | 1.04 | 1.32 | 1.51 | 1.57 |
| Blautia | 0.82 | 1.07 | 1.07 | 0.84 | 0.99 | 0.65 |
| Roseburia | 2.14 | 2.02 | 1.94 | 0.83 | 0.47 | 1.02 |
| Megasphaera | 1.69 | 1.46 | 1.4 | 1.22 | 0.91 | 2.2 |
| Lactobacillus | 1.26 | 1.06 | 1.77 | 0.81 | 1.72 | 21.2 |
| Faecalibacterium | 1.12 | 1.14 | 1.14 | 0.22 | 0.2 | 0.17 |
| Catenibacterium | 0.86 | 0.94 | 0.95 |  |  |  |
| Other | 0.76 | 0.71 | 0.78 | 0.9 | 0.84 | 0.88 |

**Supplementary Table 3.** Represents the extent of times change in microbial taxa in two different subjects due to synbiotic treatement. Data corresponds to Fig. 3G-I.

|  | **Subject A** | | | **Subject B** | | |
| --- | --- | --- | --- | --- | --- | --- |
| **Phylum** | | | | | | |
|  | **Glucose+ECN** | **Galactose+ECN** | **Starch+ECN** | **Glucose+ECN** | **Galactose+ECN** | **Starch+ECN** |
| Bacteroidetes | 0.99 | 1.31 | 1.18 | 1.02 | 0.86 | 0.7 |
| Firmicutes | 1.04 | 0.78 | 0.74 | 0.85 | 0.7 | 0.81 |
| Actinobacteria | 0.93 | 0.24 | 1.26 | 1.18 | 1.67 | 1.74 |
| Proteobacteria | 0.36 | 0.41 | 0.14 | 1.54 | 1.86 | 1.63 |
| Cyanobacteria | 0.28 | 0.28 | 0.1 | 6.89 | 7.4 | 3.51 |
| Verrucomicrobia | 0.01 | 0.02 | 0 |  |  |  |
| Tenericutes | 0.41 | 0.1 | 0.1 |  |  |  |
| TM7 | 0.62 | 0.89 | 0.48 | 0.98 | 0 | 0 |
| Fusobacteria |  |  |  | 0.74 | 0.26 | 0.88 |
| [Thermi] |  |  |  | 0 | 0.15 | 0.17 |
| Others | 0.75 | 0.29 | 0.36 | 0.98 | 0.21 | 1.41 |
| **Order** | | | | | | |
| Bacteroidales | 0.99 | 1.31 | 1.18 | 1.02 | 0.86 | 0.7 |
| Clostridiales | 1.16 | 1.02 | 0.69 | 0.86 | 0.71 | 0.82 |
| Bifidobacteriales | 0.75 | 0.27 | 1.49 | 1.07 | 1.52 | 1.87 |
| Coriobacteriales | 1.32 | 0.19 | 0.8 | 1.29 | 1.8 | 1.63 |
| Lactobacillales | 0.73 | 0.28 | 1.34 | 0.45 | 0.25 | 0.7 |
| Erysipelotrichales | 0.83 | 0.19 | 0.48 | 1.1 | 0.47 | 0.86 |
| Burkholderiales | 0.24 | 0.47 | 0.06 | 1.53 | 1.88 | 1.67 |
| Enterobacteriales | 0.73 | 0.53 | 0.38 | 2.08 | 1.81 | 1.3 |
| YS2 | 0.28 | 0.28 | 0.08 |  |  |  |
| Verrucomicrobiales | 0.01 | 0.02 | 0 |  |  |  |
| Other | 0.41 | 0.22 | 0.23 | 0.36 | 0.39 | 0.46 |
| **Genus** | | | | | | |
| Prevotella | 1.02 | 1.35 | 1.22 | 0.04 | 0.04 | 0.03 |
| Bacteroides | 0.44 | 0.5 | 0.24 | 1.03 | 0.86 | 0.7 |
| Bifidobacterium | 0.74 | 0.27 | 1.49 | 1.07 | 1.52 | 1.87 |
| Collinsella | 1.57 | 0.19 | 1.03 | 1.31 | 1.83 | 1.68 |
| Blautia | 1.31 | 0.2 | 0.56 | 0.84 | 0.84 | 0.69 |
| Roseburia | 2.49 | 4.19 | 1.6 | 0.78 | 0.45 | 0.82 |
| Megasphaera | 1.44 | 1.48 | 1.26 | 1.27 | 1.15 | 2.91 |
| Faecalibacterium | 1.44 | 0.95 | 0.8 | 0.19 | 0.16 | 0.19 |
| Lactobacillus | 0.75 | 0.29 | 1.4 | 0.74 | 0.79 | 2.78 |
| Catenibacterium | 0.87 | 0.21 | 0.53 |  |  |  |
| Others | 0.7 | 0.48 | 0.32 | 0.93 | 0.81 | 0.8 |

**Supplementary Table 4.** Represents the extent of times change in microbial taxa due to probiotic treatement. Data corresponds to Fig. 5H-J.

|  | **Ctrl vs ECN** |
| --- | --- |
|  | **Phylum** |
| Firmicutes | 1.07 |
| Bacteroidetes | 0.87 |
| Actinobacteria | 1.12 |
| Proteobacteria | 1.57 |
| Cyanobacteria | 1 |
| Verrucomicrobia | 1.9 |
| Tenericutes | 2.2 |
| TM7 | 2.17 |
| Lentisphaerae | 0.88 |
| Fusobacteria | 1.41 |
| Others | 0.95 |
|  | **Order** |
| Bacteroidales | 0.87 |
| Clostridiales | 1.05 |
| Coriobacteriales | 1.24 |
| Bifidobacteriales | 1 |
| Erysipelotrichales | 1.11 |
| Lactobacillales | 1.27 |
| Burkholderiales | 1.37 |
| Enterobacteriales | 3.73 |
| YS2 | 0.99 |
| Verrucomicrobiales | 1.9 |
| Others | 1.25 |
|  | **Genus** |
| Prevotella | 0.77 |
| Bacteroides | 1.64 |
| Bifidobacterium | 1.12 |
| Collinsella | 1.85 |
| Blautia | 1.47 |
| Roseburia | 1.33 |
| Lactobacillus | 1.29 |
| Faecalibacterium | 1.29 |
| Megasphaera | 0.99 |
| Coprococcus | 1.58 |
| Others | 1.16 |

**Supplementary Table 5.** Represents the extent of times change in microbial taxa due to prebiotic treatement. Data corresponds to Fig. 6H-J.

|  | **Control vs prebiotics** | | |
| --- | --- | --- | --- |
|  | **Glucose** | **Galactose** | **Starch** |
|  | **Phylum** | | |
| Bacteroidetes | 0.98 | 0.91 | 0.86 |
| Firmicutes | 0.94 | 0.88 | 0.97 |
| Actinobacteria | 1.17 | 1.54 | 1.44 |
| Proteobacteria | 1.34 | 1.51 | 1.54 |
| Cyanobacteria | 0.74 | 0.67 | 0.91 |
| Verrucomicrobia | 0.03 | 0.01 | 0.04 |
| Tenericutes | 0.3 | 0.52 | 1.46 |
| TM7 | 1.51 | 2.61 | 2.49 |
| Fusobacteria | 2.13 | 0 | 1.19 |
| Thermi | 0.29 | 0.16 | 0.27 |
| Others | 0.46 | 0.79 | 1 |
|  | **Order** | | |
| Bacteroidales | 0.98 | 0.91 | 0.86 |
| Clostridiales | 0.94 | 0.87 | 0.91 |
| Bifidobacteriales | 1.01 | 1.63 | 1.43 |
| Coriobacteriales | 1.35 | 1.45 | 1.44 |
| Lactobacillales | 1.13 | 0.96 | 1.64 |
| Erysipelotrichales | 0.86 | 0.92 | 0.97 |
| Burkholderiales | 1.48 | 1.71 | 1.72 |
| YS2 | 0.7 | 0.65 | 0.88 |
| Enterobacteriales | 0.67 | 0.49 | 0.61 |
| RF32 | 0.49 | 0.42 | 0.74 |
| Others | 0.37 | 0.37 | 0.7 |
|  | **Genus** | | |
| Prevotella | 0.94 | 0.92 | 0.89 |
| Bacteroides | 1.07 | 0.87 | 0.81 |
| Bifidobacterium | 1.01 | 1.63 | 1.43 |
| Collinsella | 1.39 | 1.52 | 1.5 |
| Blautia | 0.84 | 1 | 0.7 |
| Roseburia | 1.15 | 0.85 | 1.25 |
| Megasphaera | 1.56 | 1.31 | 1.63 |
| Lactobacillus | 1.26 | 1.06 | 1.81 |
| Faecalibacterium | 1.11 | 1.13 | 1.13 |
| Catenibacterium | 0.86 | 0.94 | 0.95 |
| Others | 0.83 | 0.78 | 0.84 |

**Supplementary Table 6.** Represents the extent of times change in microbial taxa due to synbiotic treatement. Data corresponds to Fig. 7H-J.

| **Control vs synbiotics** | | | |
| --- | --- | --- | --- |
|  | **Glucose+ECN** | **Galactose+ECN** | **Starch+ECN** |
|  | **Phylum** | | |
| Bacteroidetes | 1.01 | 1.13 | 0.98 |
| Firmicutes | 0.95 | 0.74 | 0.78 |
| Actinobacteria | 1.11 | 1.29 | 1.61 |
| Proteobacteria | 1.25 | 1.5 | 1.26 |
| Cyanobacteria | 0.3 | 0.3 | 0.11 |
| Verrucomicrobia | 0.01 | 0.03 | 0.01 |
| Tenericutes | 0.41 | 0.1 | 0.1 |
| TM7 | 0.66 | 0.8 | 0.44 |
| Fusobacteria | 1.58 | 0.84 | 1.7 |
| Thermi | 0 | 0.15 | 0.17 |
| Others | 0.82 | 0.26 | 0.67 |
|  | **Order** | | |
| Bacteroidales | 1.01 | 1.13 | 0.98 |
| Clostridiales | 0.98 | 0.84 | 0.76 |
| Bifidobacteriales | 0.96 | 1.09 | 1.74 |
| Coriobacteriales | 1.29 | 1.51 | 1.47 |
| Lactobacillales | 0.7 | 0.28 | 1.27 |
| Erysipelotrichales | 0.83 | 0.19 | 0.49 |
| Burkholderiales | 1.32 | 1.66 | 1.41 |
| Enterobacteriales | 1.38 | 1.14 | 0.82 |
| YS2 | 0.28 | 0.28 | 0.09 |
| Verrucomicrobiales | 0.01 | 0.03 | 0.01 |
| Others | 0.4 | 0.27 | 0.3 |
|  | Genus | | |
| Prevotella | 1.01 | 0.92 | 1.22 |
| Bacteroides | 1.03 | 0.86 | 0.7 |
| Bifidobacterium | 0.95 | 1.59 | 1.74 |
| Collinsella | 1.34 | 1.79 | 1.59 |
| Blautia | 0.9 | 0.87 | 0.67 |
| Megasphaera | 1.39 | 1.38 | 1.73 |
| Roseburia | 1.2 | 0.84 | 1.01 |
| Faecalibacterium | 1.42 | 1.13 | 0.79 |
| Lactobacillus | 0.75 | 1.06 | 1.4 |
| Catenibacterium | 0.87 | 0.94 | 0.53 |
| Others | 0.83 | 0.77 | 0.59 |

**Supplementary Table 7.** Represents the extent of times change in microbial taxa due to synbiotic treatement compared to probiotic treatement.

|  | **Glucose+ECN vs. ECN** | **Galactose+ECN vs. ECN** | **Starch+ECN vs. ECN** |
| --- | --- | --- | --- |
| **Bacteroides** | 79.05 | 65.9 | 54.11 |
| **Bifidobacterium** | 1.24 | 2.06 | 2.26 |
| **Blautia** | 2.51 | 2.43 | 1.88 |
| **Collinsella** | 2.61 | 3.49 | 3.09 |
| **Faecalibacterium** | 0.56 | 0.44 | 0.31 |
| **Lactobacillus** | 0.29 | 0.41 | 0.55 |
| **Megasphaera** | 0.98 | 0.96 | 1.21 |
| **Others** | 0.66 | 0.61 | 0.47 |
| **Prevotella** | 0.66 | 0.6 | 0.8 |
| **Roseburia** | 1.83 | 1.27 | 1.54 |

**Supplementary Table 8.** Represents the extent of times change in microbial taxa due to synbiotic treatement compared to prebiotic treatement.

|  | **Glucose+ECN vs. Glucose** | **Galactose+ECN vs. Galactose** | **Starch+ECN vs. Starch** |
| --- | --- | --- | --- |
| **Bacteroides** | 0.96 | 0.98 | 0.87 |
| **Bifidobacterium** | 0.94 | 0.97 | 1.22 |
| **Blautia** | 1.07 | 0.87 | 0.96 |
| **Catenibacterium** | 1.02 | 1 | 0.56 |
| **Collinsella** | 0.97 | 1.18 | 1.06 |
| **Faecalibacterium** | 1.28 | 1 | 0.7 |
| **Lactobacillus** | 0.59 | 1 | 0.77 |
| **Megasphaera** | 0.9 | 1.05 | 1.06 |
| **Others** | 0.99 | 0.98 | 0.7 |
| **Prevotella** | 1.08 | 1 | 1.37 |
| **Roseburia** | 1.04 | 0.99 | 0.81 |


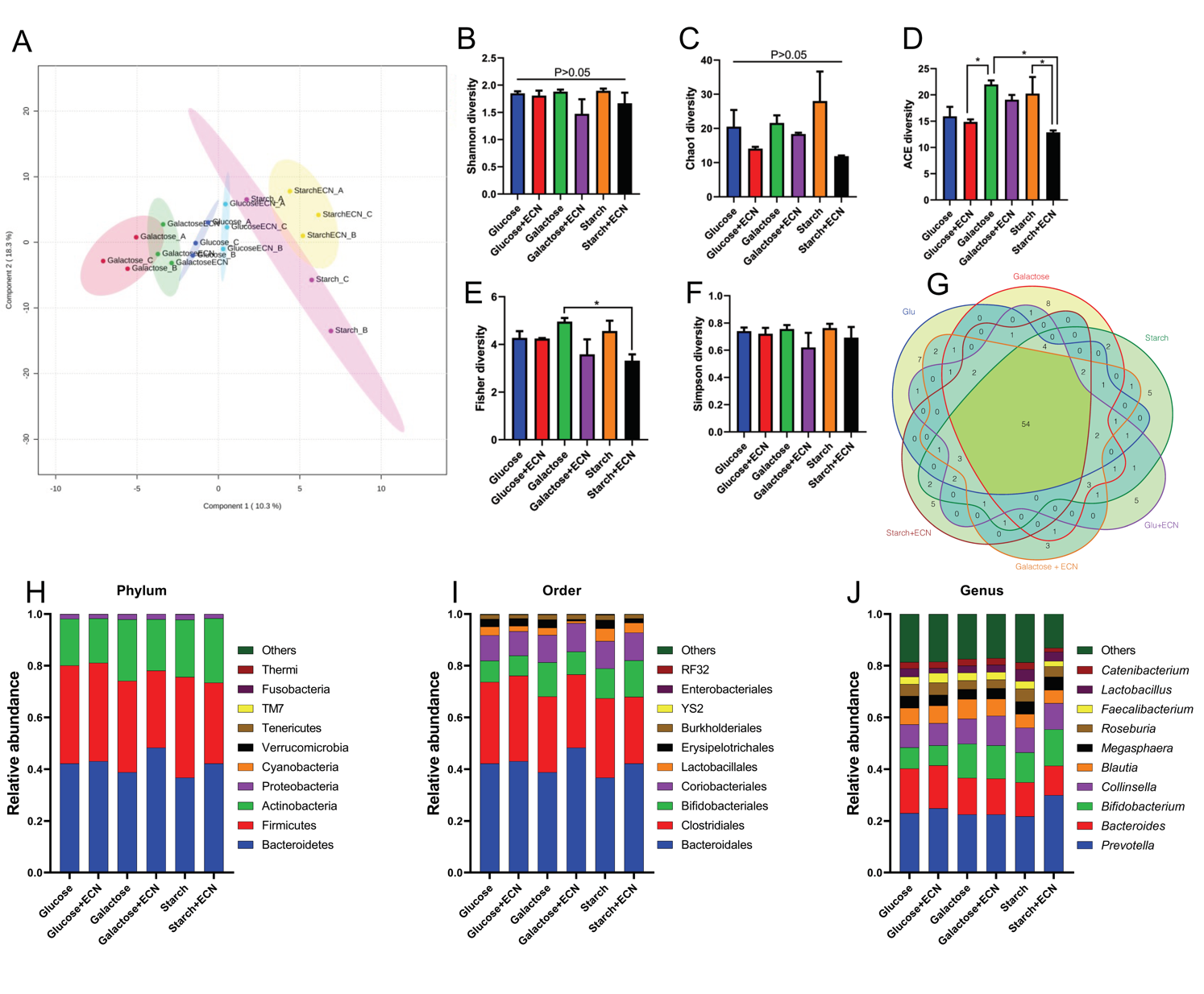


**Supplementary Fig 1.**

**Comparison of the gut microbiome modulatory effects between prebiotic (glucose, galactose or starch) and synbiotics (ECN in combination with either glucose, galactose or starch). (A)** Partial least squares-discriminant analysis (PLSDA) reveals discriminating characteristics of microbiota segregating groups based on beta-diversity. **(B-F)** Various gut microbial alpha-diversity metrics indicating variability in microbial community composition. **(G)** Venn diagram depicting commonality and uniqueness of identified genera between groups. **(H-J)** Abundance of taxa at phylum, order and genus level represented as proportions of 1.


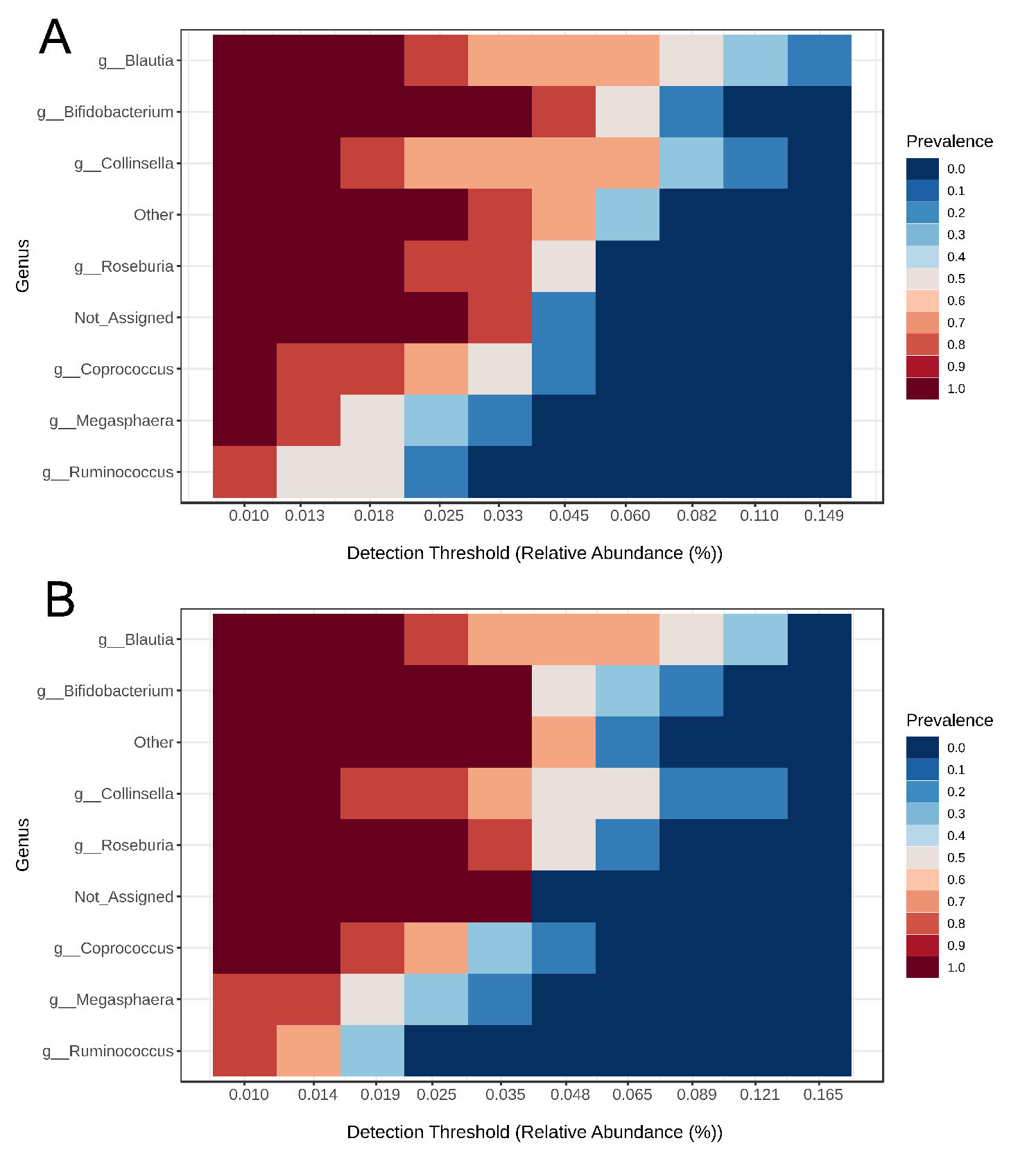


**Supplementary Fig 2.** CORE microbiome at 80% prevalence across samples of **(A)** untreated control and **(B)** under ECN-treatment under genus level. Data corresponds to Fig. 5.


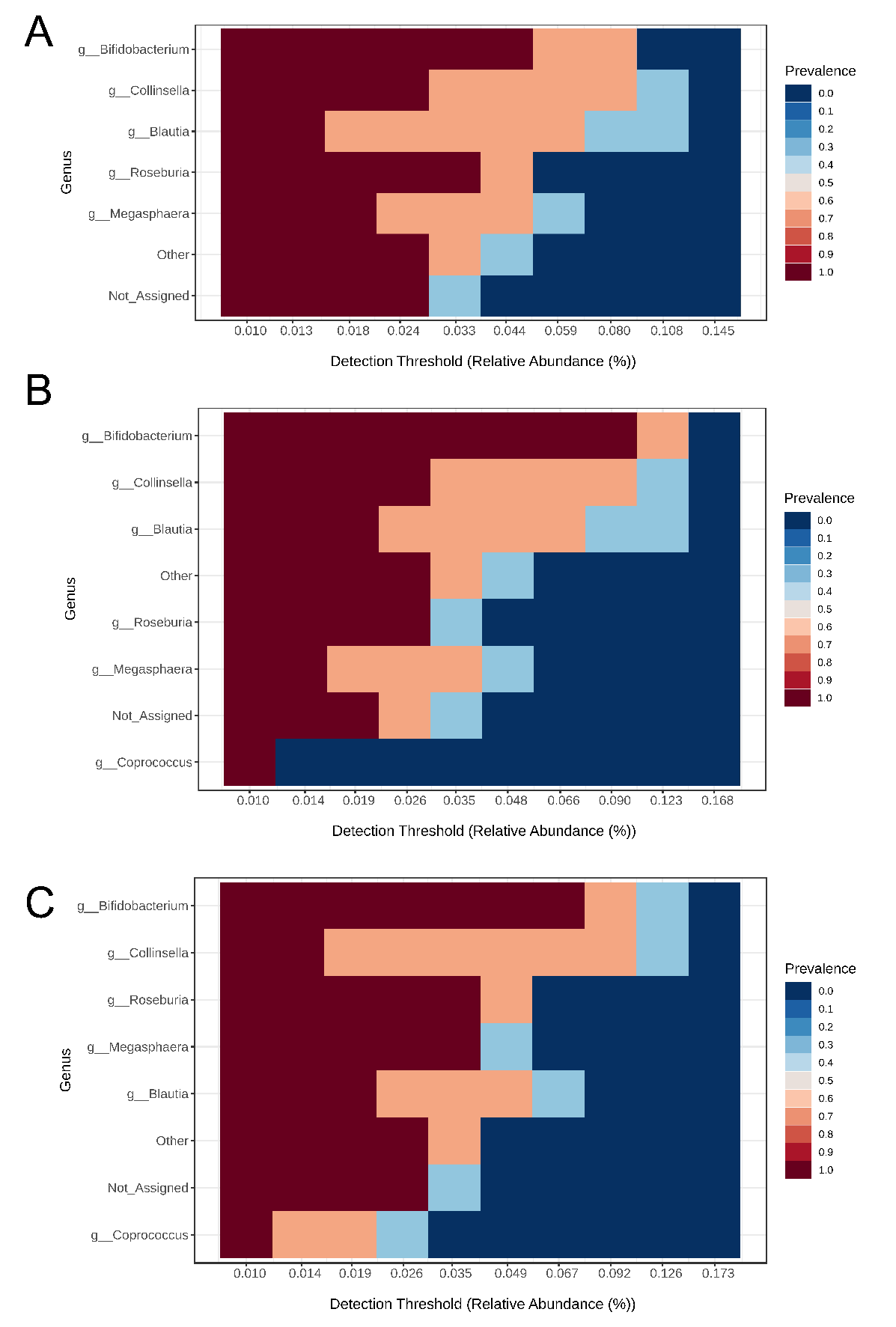


**Supplementary Fig 3.** CORE microbiome at 80% prevalence across prebiotic-treated groups at genus level. **(A)** Glucose, **(B)** Galactose, and **(C)** Starch. Data corresponds to Fig. 6.


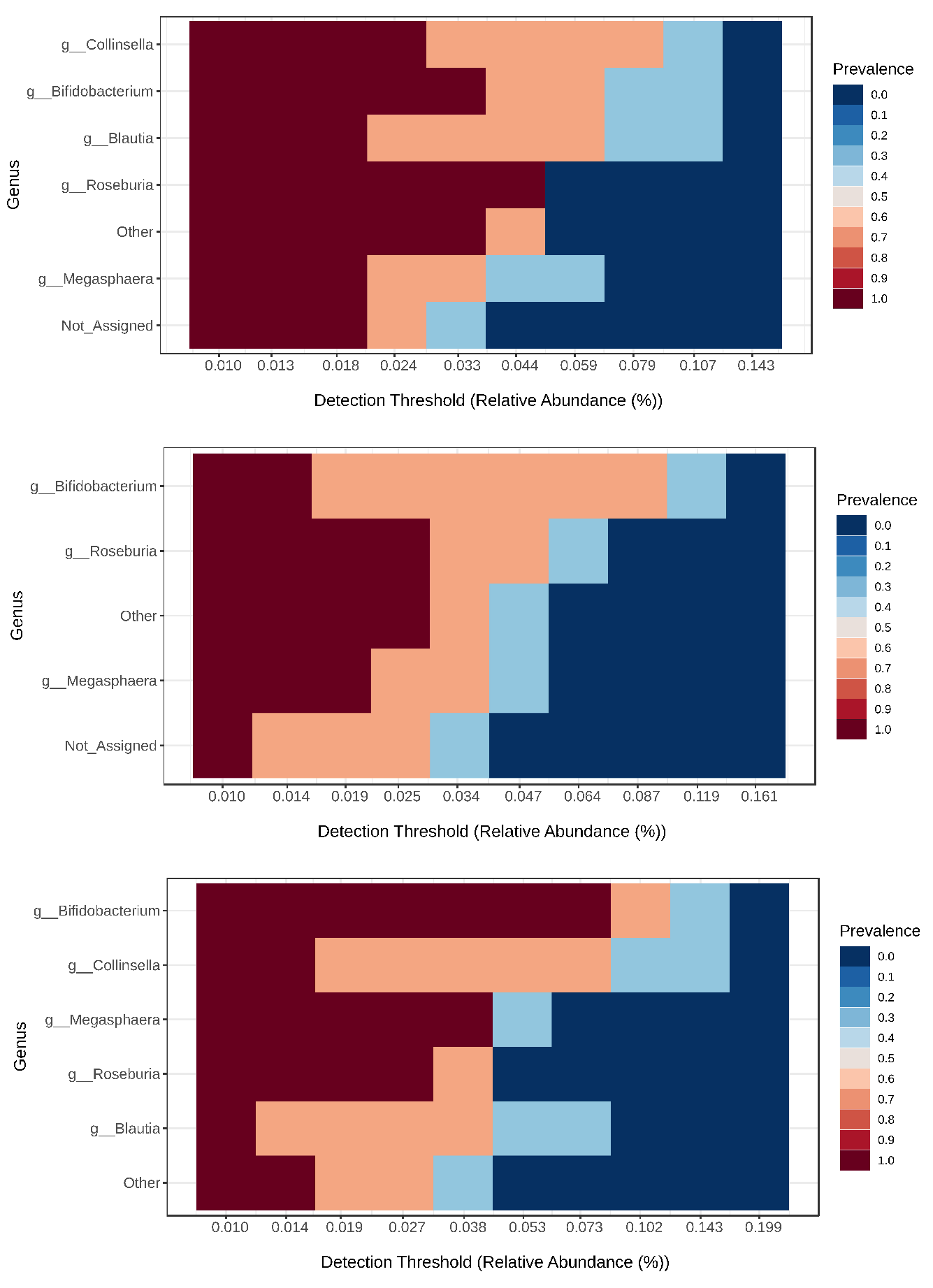
**Supplementary Fig 4.** CORE microbiome at 80% prevalence across synbiotic-treated groups at genus level. **(A)** ECN+Glucose, **(B)** ECN+Galactose, and **(C)** ECN+Starch. Data corresponds to Fig. 7.
